# Thrombospondin-1 Contributes to Mortality in Murine Sepsis through Effects on Innate Immunity

**DOI:** 10.1371/journal.pone.0019654

**Published:** 2011-05-09

**Authors:** Sara McMaken, Matthew C. Exline, Payal Mehta, Melissa Piper, Yijie Wang, Sara N. Fischer, Christie A. Newland, Carrie A. Schrader, Shannon R. Balser, Anasuya Sarkar, Christopher P. Baran, Clay B. Marsh, Charles H. Cook, Gary S. Phillips, Naeem A. Ali

**Affiliations:** 1 Division of Pulmonary, Allergy, Critical Care, and Sleep Medicine, Department of Internal Medicine, The Dorothy M. Davis Heart and Lung Research Institute, Ohio State University, Columbus, Ohio, United States of America; 2 Department of Surgery, Ohio State University, Columbus, Ohio, United States of America; 3 The Center for Biostatistics, Ohio State University, Columbus, Ohio, United States of America; University of Minnesota, United States of America

## Abstract

**Background:**

Thrombospondin-1 (TSP-1) is involved in many biological processes, including immune and tissue injury response, but its role in sepsis is unknown. Cell surface expression of TSP-1 on platelets is increased in sepsis and could activate the anti-inflammatory cytokine transforming growth factor beta (TGFβ1) affecting outcome. Because of these observations we sought to determine the importance of TSP-1 in sepsis.

**Methodology/Principal Findings:**

We performed studies on TSP-1 null and wild type (WT) C57BL/6J mice to determine the importance of TSP-1 in sepsis. We utilized the cecal ligation puncture (CLP) and intraperitoneal *E.coli* injection (IP *E.coli*) models of peritoneal sepsis. Additionally, bone-marrow-derived macrophages (BMMs) were used to determine phagocytic activity. TSP-1−/− animals experienced lower mortality than WT mice after CLP. Tissue and peritoneal lavage TGFβ1 levels were unchanged between animals of each genotype. In addition, there is no difference between the levels of major innate cytokines between the two groups of animals. PLF from WT mice contained a greater bacterial load than TSP-1−/− mice after CLP. The survival advantage for TSP-1−/− animals persisted when IP *E.coli* injections were performed. TSP-1−/− BMMs had increased phagocytic capacity compared to WT.

**Conclusions:**

TSP-1 deficiency was protective in two murine models of peritoneal sepsis, independent of TGFβ1 activation. Our studies suggest TSP-1 expression is associated with decreased phagocytosis and possibly bacterial clearance, leading to increased peritoneal inflammation and mortality in WT mice. These data support the contention that TSP-1 should be more fully explored in the human condition.

## Introduction

Severe sepsis and septic shock are conditions that affect millions every year, killing approximately 30% of those afflicted [1]. By recent projections the incidence and morbidity of sepsis are increasing 1–3. This clinical syndrome is believed to be triggered by bacterial products released from a previously localized infection that leads to a systemic inflammatory reaction and organ impairment [4]. These triggers drive organ failure and worse outcomes potentially through dysregulation of the immune response, activation of platelets and coagulation cascades which propagates the inflammatory response [5]. Of the various sepsis syndromes, peritoneal sepsis due to visceral pathology or spontaneous bacterial translocation has a very high mortality rate of 60–80% [2], [3], [6].

TSP-1 is a matrix glycoprotein with diverse roles in numerous cellular and physiologic processes [7]. The primary source of TSP-1 is activated platelets, where it comprises 25% of the total protein content of its alpha-granules [8]. Other tissues and cells including leukocytes and endothelial cells can also produce this matricellular protein [8]–[10]. Platelet surface expression of TSP-1 is increased in patients with sepsis, and polymorphisms in TSP-1 are associated with developing sepsis-related organ failure [11], [12]. TSP-1 expression is thought to play an important role in multiple biological processes including thrombosis, wound healing and the immune response [13]–[16]. It can influence the regulation of innate immune functions directly through its effects on phagocytic cell migration and indirectly through activation of the potent anti-inflammatory cytokine transforming growth factor beta (TGFβ1) [17], [18]. TGFβ1 has been shown to be important to the development of sepsis-related organ failure and the inflammatory response to infections [19], [20].

The immune system's ability to contain local infections is an important part of the normal response to infection. In peritoneal sepsis related to disrupted bowel integrity, local wound healing and bacterial clearance are important components of this response [21]. The influx of plasma proteins and activation of the clotting cascade can begin to locally control injury, but controlled inflammation drives further cellular and cytokine responses leading to wound healing [21]. Prior studies have demonstrated that fibroblasts[22]–[24], collagen production [25], and fibrogenic growth factors (Connective tissue growth factor, CTGF; TGFβ1; Vascular endothelial growth factor, VEGF) [15], [16], [26], [27] are important in this process. Additionally, the recruitment of phagocytes is critical to this local bacterial clearance [28]. When microorganisms cannot be controlled locally, bacteremia can result [4], [5].

A potential role for TSP-1 in the pathogenesis of sepsis has not been previously shown. Our primary aim was to determine if survival in murine severe sepsis was affected by TSP-1 status. We hypothesized that TSP-1 could influence sepsis outcome through either an effect on local wound healing at the site of surgical injury after cecal ligation and puncture (CLP) or bacterial clearance. Here, we demonstrate that mice deficient in TSP-1 have improved survival and bacterial clearance in murine models of sepsis.

## Results

### TSP-1−/− mice are protected from CLP-related mortality and experience lower peritoneal bacterial load despite equal cecal injury

**Figure 1:**
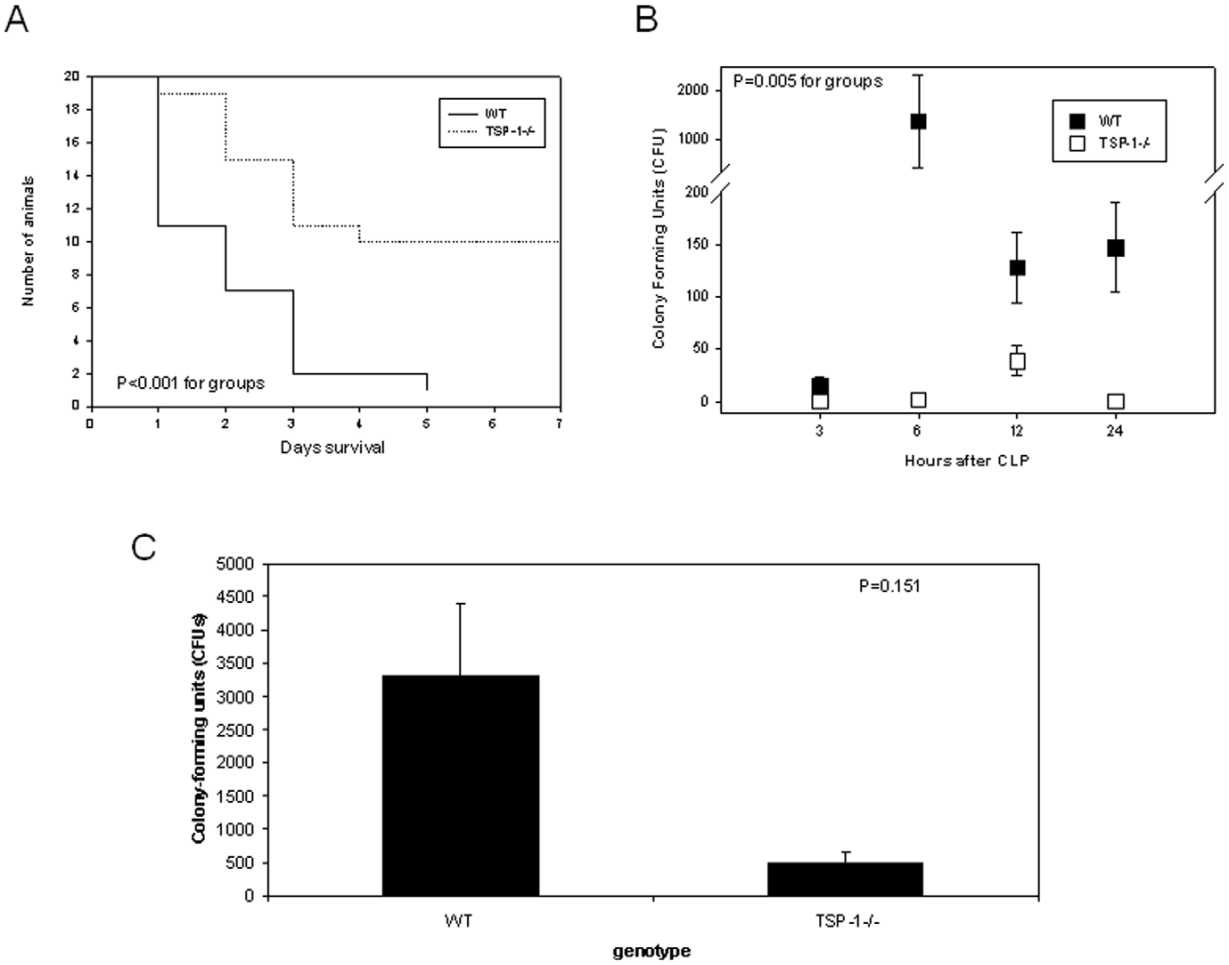
TSP-1 deficient mice are resistant to surgical sepsis-induced mortality, and have a lower peritoneal bacterial load following induction of surgical peritonitis. WT and TSP-1−/− mice were subjected to either CLP or sham surgery as described in Materials and Methods on Day 0. A. Mice were monitored for a course of 7 days for survival (p<0.001 by Kaplan-Meier, n = 20 WT CLP, n = 20 TSP-1−/− CLP). All mice receiving sham surgeries survived until study end (7 days) (n = 5 WT sham, n = 6 TSP-1−/−sham). B. Mice underwent CLP surgery at hour 0 and were euthanized at various time points after surgery. Sterile peritoneal lavage was performed and colony-forming unit counts were performed on the lavage fluid from mice 3, 6, 12, and 24 hours after the CLP procedure. Data represents 3 mice per time point, and error bars are +/−SEM (*p<0.05, for 6, 12 and 24 hours after CLP, ANOVA). C. Spleens were harvested under sterile conditions from WT and TSP-1−/−mice 3 hours post-CLP. Organs were then crushed under sterile conditions. Data represents colony-forming unit counts from total organ protein of 3 mice per genotype and error bars are +/−SEM (*p = 0.04).

We induced polymicrobial sepsis in WT and TSP-1 −/− mice via cecal-ligation and puncture (CLP) that results in reproducible inflammatory injury [29]. WT mice had an observed mortality of 95% within 72 hours of CLP, a rate significantly higher than TSP-1−/− mice (Figure 1A). Sham operation resulted in 100% seven-day survival regardless of genotype. To characterize the intra-abdominal response, peritoneal lavage fluid (PLF) was analyzed for cellular composition, inflammatory cytokines and bacterial load. TSP-1 −/− and WT mice developed similar peritoneal cellular responses after CLP (Table 1), but there was a significantly higher peritoneal bacteria count observed in WT mice after CLP (Figure 1B). In addition, WT animals and TSP-1 −/− animals had no significant differences in PLF KC (IL-8), Tumor necrosis factor-a, IL-6 or IL-1b for up to 24 hours after CLP (n = 3 for each background and time point, 6, 12 and 24 hours after CLP). Serum inflammatory cytokines (IL-1b, IL-6, TNF-alpha, KC and HMGB1) were not significantly different 12 hours after CLP (Table 2). Peritoneal bacterial burden correlated well with bacterial quantity in cultured splenic homogenates, suggesting worsened bacteremia in WT mice (Figure 1C). Based on these findings, we sought to understand why local control of peritoneal infection was enhanced in TSP-1 −/− mice.

**Table 1:** Peritoneal lavage fluid cell counts after CLP.

	% of total			
Parameter	Total Cells	Neutrophils	Macrophages	Lymphocytes
WT 3 hrs	6.0×10^5^±1.1×10^5^	95±0.6	3.3±0.3	1.7±0.7
TSP-1−/− 3 hrs	8.3×10^5^±3.3×10^5^	96±0.3	3.0±0.1	1.3±0.3
WT 6 hrs	3.9×10^6^±4.0×10^5^	90±0.6	8.3±0.9	1.7±0.3
TSP-1−/− 6 hrs	3.3×10^6^± 9.6×10^5^	89±0.7	8.7±0.3	2.0±0.6
WT 12 hrs	1.9×10^7^±6.6×10^6^	64±0.3	33±1.0	3.3±0.9
TSP-1−/− 12 hrs	1.1×10^7^±4.9×10^6^	65±1.2	32±1.0	3.0±0.6

Data represent mean ± SEM. n = 3 for WT at 3,6,12 hours, n = 3 for TSP-1−/− at 3,6,12 hours.

**Table 2:** Serum pro-inflammatory cytokine analysis.

	TSP deficiency	WT	p-value
**VEGF**	105.3 (67.7–117.6)	193.3 (90.2–245.9)	0.18
**IL-1beta**	342.1 (108.5–557.8)	394.0 (378.1–795.3)	0.16
**IL-6**	603.3 (230.2–685.5)	1,577.1 (596.7–1788.7)	0.46
**KC**	2,347.6 (1707.4–3800.1)	3,029.9 (2324.9–3734.9)	1.00
**TNF-alpha**	289.9 (223.9–319.2)	323.6 (263.5–712.2)	0.21
**HMGB1**	17.5 (11.5–26.3)	62.5 (4.9–230.0)	0.61

Data represent median (25^th^–75^th^ percentile). n = 9 for WT or TSP deficient animals at 12 hours after CLP; all pg/ml except HMGB1 in ng/ml.

### TSP-1−/− macrophages have increased phagocytic capacity and are protected from mortality in a non-surgical infectious model of peritoneal sepsis

**Figure 2:**
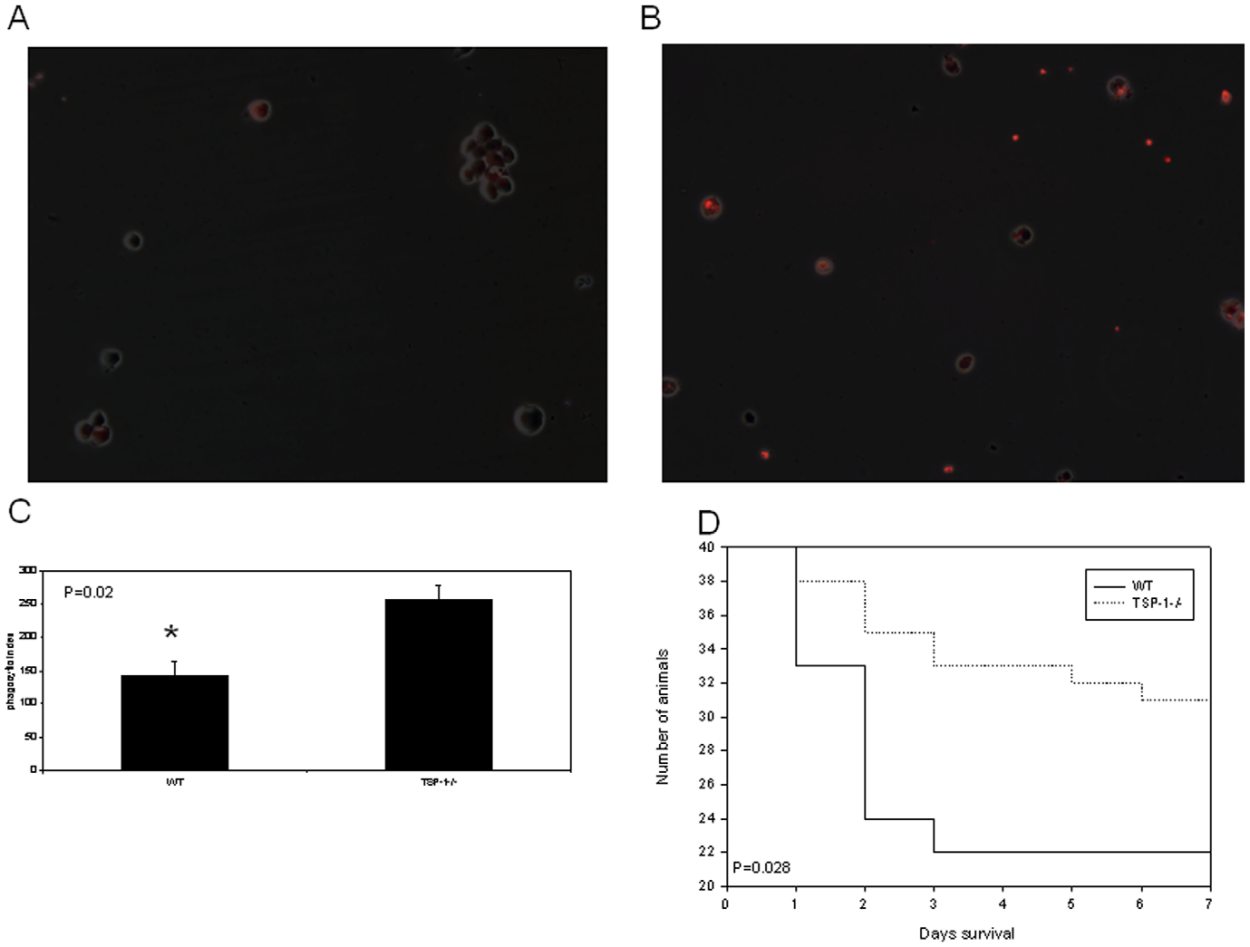
Mice containing functional TSP-1 protein have decreased phagocytic capacity as compared to mice lacking functional TSP-1, and show increased survival in a non-surgical model of sepsis. BMMs were co-incubated with IgG-opsonized, labeled sheep red blood cells and phagocytic index was determined via blinded counting of ingested red blood cells per 100 macrophages. Phase contrast images of BMMs overlayed with images of SRBCs ingested are shown for WT mice (A) and TSP-1−/− mice (B). C. The quantification of phagocytic capacity is indicated by phagocytic index. Images and Data represent n = 3 for WT animals and n = 3 for TSP-1−/− animals, and error bars are +/– SEM (*p<0.01 by student's t test). D. WT and TSP-1−/− mice were subjected to either *E.coli* or PBS injection as described in Materials and Methods on Day 0. Mice were monitored for a course of 7 days for survival (p<0.001 by Kaplan-Meier, n = 40 WT CLP, n = 40 TSP-1−/− *e.coli* injection). All mice receiving sham surgeries lived the course of 7 days (n = 5 WT PBS, n = 5 TSP-1−/− PBS).

As stated earlier, the number and type of inflammatory cells (Table 1) and PLF pro-inflammatory cytokines were similar between WT and TSP-1−/− mice, therefore differences in phagocytic function could lead to changes in bacterial clearance and therefore quantity at fixed times after contamination. Using BMMs in a well-defined phagocytosis assay [30], we observed that TSP-1−/− macrophages ingested 70% higher amounts of opsonized SRBCs than WT (Figure 2A–C). This differences in activity could not be explained by the proportion of cells expressing phagocytic markers (CD64) on all peritoneal cells (3.0% TSP−/− vs. 4.8% WT, p = 0.22) or only those co-expressing macrophage markers (F4/80) (20.7% TSP−/− vs. 22.8% WT, p = 0.71). To determine the relevance of these *in vitro* findings, we performed survival studies between the two backgrounds in a purely infectious model of peritonitis. This approach minimizes the possibility that mortality is related to surgical wound healing or variation in fecal contamination of the peritoneum, while still investigating bacterial contamination of the peritoneum. We injected 5×10^9^ CFU/ kg *E.coli* IP to LD50 for WT and TSP-1−/− mice [31]. Survival for TSP-1−/− mice was significantly higher than that observed in WT after *E.coli* injection (p = 0.028, Figure 2D).

### TSP-1 deficient and WT mice exhibit similar wound healing after CLP

**Figure 3:**
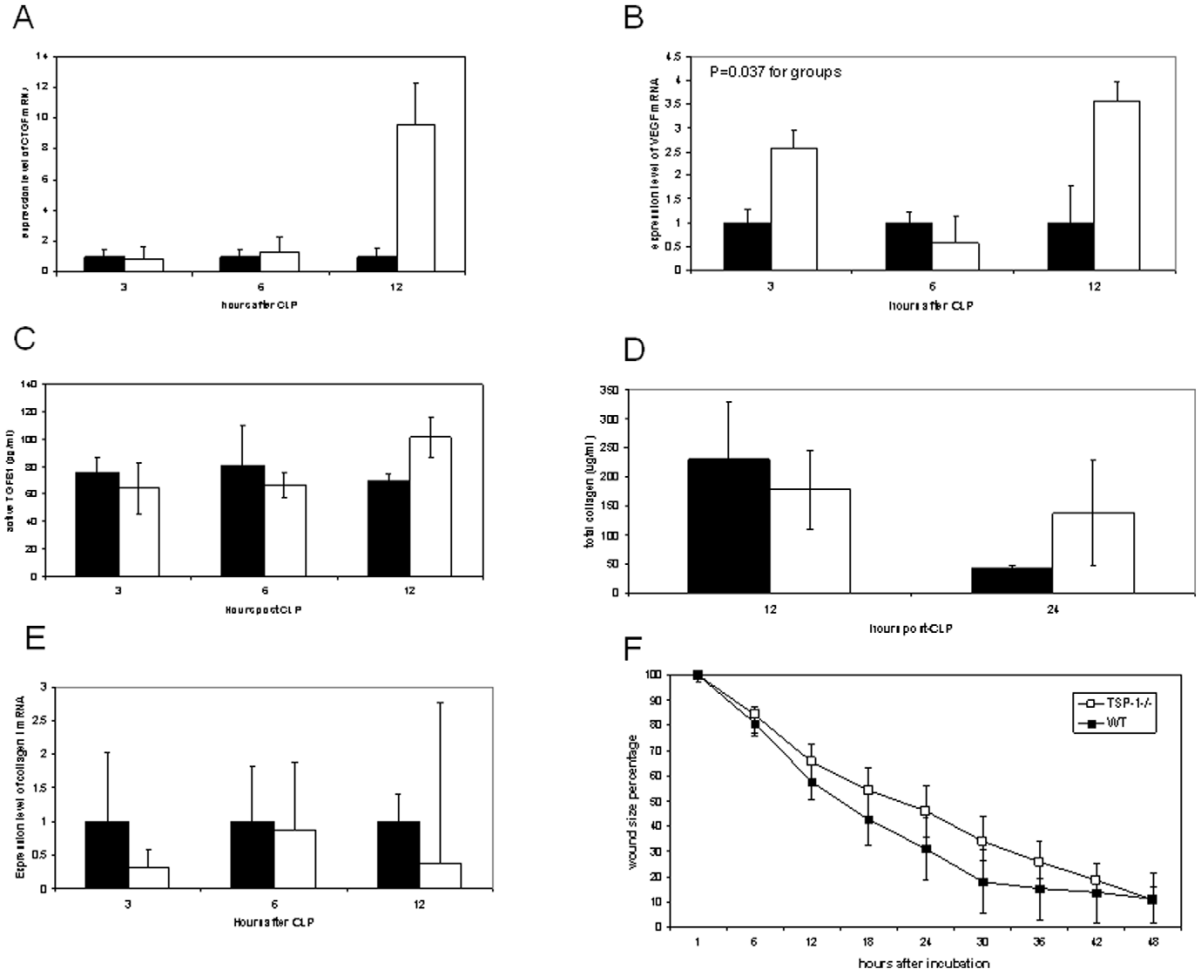
TSP-1 deficient and WT mice exhibit similar amounts of wound healing markers after CLP. CLP surgery was performed on WT and TSP-1−/− mice, and cecums were harvested 3, 6, and 12 hours afterwards and immediately processed for protein and RNA assessment [CTGF (A) and VEGF (B) and collagen I (E)]. [For all panels, WT (black bars) and TSP-1−/− (white bars); error bars represent ±SEM] A. Cecal CTGF transcript is shown for each timepoint after surgery. Data represents n = 6 for WT and TSP-1−/− at each timepoint. B. Cecal VEGF transcript is shown for each timepoint after surgery. Data represents n = 5 for 3 hours, n = 6 for 6 hours and n = 3 for 12 hours. C. Cecal protein lysates were assessed via ELISA for active TGFβ1. Data represents active TGFβ1 in 50 µg total protein/sample (n = 6). D. Cecums were harvested at 12 and 24 hours after CLP and collagen measured via Sircoll Assay. Data represents n = 3 for all timepoints. E. Cecal collagen I transcript is shown for each timepoint after surgery. Data represents n = 6 for background and timepoint. F. WT and TSP-1 −/− fibroblasts were compared in their ability to close a mechanical scratch *in vitro* by scratch closure assay. The mechanical defect was photographed hourly for 48 hours, and unclosed scratch area (“wound” size) was determined using Photoshop. Data points represent the “wound” size at each time point for each background [n = 4 for WT (black squares) and n = 5 for TSP-1−/− (white squares)].

Because TSP-1 is known to be involved in epithelial wound healing [32] and CLP is a surgical injury that requires healing for survival, a separate cohort was studied to determine the effect of TSP-1 status on cecal wound healing. We assayed ceca from CLP in TSP-1−/− and WT mice for TGFβ, CTGF and VEGF. We did not find a significant difference in CTGF or TGFβ protein at any time point within the first 24 hours after CLP (Figure 3A, C). VEGF mRNA was statistically different from WT expression after CLP with the point estimate being higher at the 3 and 12 hour timepoint, but lower at 6 hours in TSP-1−/− compared to WT (Figure 3B). No differences in the amount of peritoneal lavage fluid TGFβ1 protein were observed between WT and TSP-1−/− animals.

To confirm the significance of our fibrogenic growth factor assays, collagen I protein and mRNA were measured. Neither of these parameters was affected 12 or 24 hours after injury by the presence of functional TSP-1 (Figure 3D and 3E). Sham-treated cecal tissues had no detectable expression of collagen protein or message (data not shown). Finally, in a fibroblast scratch assay TSP-1−/− fibroblasts showed normal mobility relative to WT fibroblasts, suggesting one critical aspect of wound closure is not different between the genotypes (Figure 3F).

## Discussion

The data presented in this report highlights the importance of TSP-1 in macrophage phagocytosis, bacterial clearance, and outcome in two models of murine sepsis. Taken together, this suggests that TSP-1 expression could impact outcome through a negative effect on innate immune function in patients with severe sepsis. Given that murine TSP-1 deficiency is associated with increased circulating and infiltrating leukocytes [33], our observations suggest that TSP-1 could serve to both reduce leukocyte infiltration and its phagocytic function.

Previous studies have suggested that TSP-1's role in inflammation is due to TGFβ1 activation. TSP-1 has been shown to activate latent TGFβ1 *in vitro*, and TSP-1−/− mice recapitulate many of the same phenotypes that TGFβ1−/− mice display [33]. Thus, it is believed that TSP-1 can activate TGFβ1 *in vivo* [34]. More recent data suggests that interaction of the LAP-binding integrins αvβ6 and αvβ8 with latent TGFβ1 may play a more central role in activation [35]. Additionally, LAP may independently utilize TSP-1 interactions to induce a biologic response [17], suggesting that other roles for TSP-1 could be more important than activation of TGFβ1. In peritoneal sepsis, it appears that non-TSP related mechanisms activate TGFβ1, as levels of the active protein localized to the area of injury (ceca) or surrounding environment (peritoneal lavage fluid) were unchanged in TSP-1−/− mice compared to WT mice after CLP. Additionally, there appeared to be no differences in peritoneal lavage fluid pro-inflammatory cytokines after CLP, suggesting, in part, that TSP-1's role in the pathogenesis of surgical sepsis is separate from its ability to activate TGFβ1.

In tumor microenvironment studies, TSP-1 can act as a monocyte chemoattractant [17], [36], [37]. Yet, we observed no difference in the number or type of leukocytes recruited after CLP between TSP-1−/− and WT mice. Previous reports have implicated TSP-1 expression as essential to phagocytosis of apoptotic fibroblasts and neutrophils, but little is known about TSP-1 role in response to bacterial challenge [38], [39]. Our data suggests that TSP-1 deficiency confers increased viability after septic insult by upregulating innate immune responsiveness leading to more rapid clearance of peritoneal bacteria without effecting cytokine production. We show that murine macrophages lacking TSP-1 display an increased capacity for FcγR-mediated phagocytosis. This phagocytic difference appears relevant as it is supported by increased survival in TSP-1−/− mice compared to WT mice after isolated bacterial challenge. The mechanism whereby TSP-1 directly influences phagocytic function is being actively pursued in our laboratory, but at present is unknown.

These findings are made more relevant by the observation that TSP-1 expression can be observed in critically ill patients with severe sepsis [11]. While it is possible this is only secondary to platelet activation, there may be some biologic importance to its elevation during sepsis given the demonstrated effect on phagocytic function in the current report. The susceptibility of critically ill patients to nosocomial infections is well described [40], yet its causes are not completely delineated. Because critically ill and septic patients have been demonstrated to have reduced phagocytic function [40], [41], it is possible that TSP-1 mediates at least part of this response.

There are several limitations to our studies. First we have only presented several select important measures of wound healing in our analysis and our number of animals could lead us to falsely concluding no significant difference exists in these markers. While this is remediable, we believe the concordance of the observations and the early mortality in these animals make it unlikely that either lavage inflammation or wound healing factors are likely to play an important role in larger studies. Secondly, we have not defined the precise mechanisms whereby the absence of TSP-1 leads to increased bacterial clearance. While cell counts and differentials are similar between TSP-1 −/− and WT animals undergoing CLP, it is possible that the activation or phagocytic state is altered and this remains undefined. Thirdly, we do not have data to demonstrate that colonic bacterial colonization is equal in each genetic background. This could lead to higher intraperitoneal bacterial count between the two backgrounds with equal fecal spillage. While this is possible, the presence of similar survival advantage when using a non-surgical method for peritoneal sepsis (IP live *E. coli* injection) in TSP-1 −/− animals where fecal spillage does not play a role and standard inoculums are injected makes this unlikely. Finally, our observations in knockout animals were not supplemented with a transgenic animal model and as a result we do not know if true “over-expression” would yield similar results. We hope to explore these further in more dedicated human studies in the future to add to the relevance of the murine observations.

In summary, we demonstrate that the presence of TSP-1 in murine sepsis is associated with increased mortality and reduced bacterial clearance. This observation is not explained by TSP-1's well-characterized role in wound healing, but rather by an associated negative regulation of innate immune cell phagocytic capacity. These observations deserve further investigation in human peritoneal and non-peritoneal sepsis.

## Materials and Methods

### Ethics Statement

All animal experiments were designed in accordance with the expectations of and approved in advance by The Ohio State University Institutional Animal Care and Use Committee. All procedures for experiments and monitoring for suffering were generated and applied after Veterinarian approval through this committee in accordance with international standards. This protocol was approved under application number 2009A0124.

### Mouse cecal-ligation and puncture (CLP) and peritoneal inoculation studies

Male mice aged 30–60 days on the C57BL/6J background were used for these experiments. TSP-1−/− mice were a kind gift of Dr. Jack Lawler (Harvard Medical School). These mice were generated by placing a neo insertion in exons 2–3 that disrupts ability to make functional TSP-1 protein, as previously published [36]. All mice were maintained in pathogen-free conditions and provided with food and water. For CLP experiments, mice (n = 20 per group) were anesthetized using 2% isoflurane, and a midline abdominal incision was made exposing the peritoneal cavity. One cm of the cecum was ligated using braided silk suture (Deknatal, Research Triangle Park, N.C.), and a sterile 22 gauge needle (B–D, Franklin Lakes, N.J.) was used to make 4 puncture holes in the ligated cecum. The original incision was closed using Dexon II absorbable sutures (Syneture, Norwalk, CT) and VetBond Tissue Adhesive (3M, St. Paul, MN). Sham surgeries were performed using the same abdominal incision and closure methods, but ceca were not ligated or punctured. The peritoneal injection of *E.coli* to induce sepsis was performed as previously described.[30] Briefly, mice (n = 40 per group) were injected with 5×10^9^ colony-forming units (CFU)/kg *E.coli* strain BL21DE3/kg weight resuspended in sterile PBS [31]. Control mice were injected with sterile PBS only. Survival was monitored for both techniques over the course of 7 days. Blinded personnel monitored mice for signs of distress. When mice appeared to be in extreme distress they were euthanized. Euthanized mice are included in all observed survival data.

### Organ collection and protein/RNA isolation

In experiments separate from the survival studies, mice (n≥3 animals per group) were euthanized at specific times after surgical procedure. We planned for *at least* three animals per group, but to ensure at least three animals per group survived to the appropriate endpoint, six animals underwent treatment. As a result of death before the specified timepoint the actual measured n was between three and six. Cecal tissue just distal to the ligation point from CLP-treated mice was compared to tissue 1 cm above the cecal tip from sham treated mice. Cecal tissues were either flash-frozen or flushed with sterile PBS and placed in 10% formalin for immunohistochemistry. Organs were crushed using a tissue homogenizer (Kontes Glass Co., Vineland, N.J.). Protein was extracted using Cell Signaling Lysis Buffer (Cell Signaling Technology, Danvers, MA). RNA was extracted using the Trizol method (Invitrogen, Carlsbad, CA).

#### Quantitative Real-Time PCR

mRNA was converted to cDNA using SuperScript III reagents (Invitrogen). Quantitative real-time PCR for VEGF, type I collagen, CTGF, TSP-1 and GAPDH. Endogenous control was performed using SYBR Green I reagents (Eurogentech NA, San Diego, CA) on a 7900HT Fast Real-Time PCR System (Applied Biosystems, Foster City, CA). All primers were designed using Primer Express software (Applied Biosystems). Primer sequences: mouse VEGF: 5′-TTACTGCTGTACCTCCACC-3′ (forward), 5′-ACAGGACGGCTTGAAGATG-3′ (reverse); mouse type I collagen: 5′-ATGGATTCCCGTTCGAGTACG-3′ (forward), 5′-TCAGCTGGATAGCGACATCG-3′ (reverse); mouse CTGF: 5′-AAAGTGCATCCGGACACCTAA-3′ (forward), 5′-TGCAGCCAGAAAGCTCAAACT-3′ (reverse); mouse GAPDH: 5′-GCACAGTCAAGGCCGAGAAT-3′ (forward); 5′-GCCTTCTCCATGGTGGTGAA-3′ (reverse).

### Protein Assays

Homogenized ceca (n≥3 animals per group) were assayed for active TGFβ1 via ELISA (R&D Systems, Minneapolis, MN). For measurement of collagen, mouse ceca were homogenized in 1.5 ml of 0.5 M acetic acid and rocked overnight at 4°C. Samples were centrifuged for 10 minutes at 2,000 rpm, and the resulting supernatant was assessed for collagen according to the manufacturer's instructions (Biocolor Ltd., Belfast, Northern Ireland). Cytokine assays were performed using the BioRad Bioplex murine specific inflammatory cytokine panel (BioRad, Hercules, CA) for all cytokines except HMGB1. HMGB1 assays were performed on a dedicated ELISA kit and performed on mouse serum to manufacturer specifications (IBL International GMBH, Hamburg, Germany).

### Lavage Collection and Analysis

Immediately following sacrifice, the peritoneal cavity of mice (n≥3 animals per group) was washed using 5 ml sterile PBS and 10 ml syringe fitted with catheter tube and needle. To determine amount of bacterial load from the peritoneal cavity, total lavage fluid was plated on chocolate agar and incubated overnight at 37°C and quantified as colony forming units (CFU). Total fluid was then spun in a clinical centrifuge at 1700 RPM and fluid portion was frozen at −20°C. To determine active TGFβ1, ELISA assays were performed (R&D Systems, Minneapolis, MN). Analysis was done using an EL_x_808 Microplate Reader (Bio-Tek Instruments, Winooski, VT). A standard curve *R* ^2^ value of ≥0.98 was considered acceptable for sample analysis. Cellular portion was resuspended in PBS. Total cell amount was counted using trypan blue and a hemacytometer. Cell differentials were determined using a Cytospin apparatus and Diffquick staining.

### Fibroblast Scratch Assays

2 cm of the distal ends of mouse ceca were obtained from WT and TSP-1−/− mice for fibroblast isolation. Colons were washed 2 times in PBS, incubated in 0.04% NaOH for 30 min, then washed an additional 2 times in PBS. Cleaned colon sections were then minced and placed in DMEM/10% FBS (Invitrogen) and incubated for 2 weeks at 37C with washing and media changes every 2 days to obtain primary fibroblasts. Fibroblasts were plated in 12-well plates and allowed to reach 80% confluency for scratch assays. Wells were scratched with a 10 µL pipet tip and washed 2 times with PBS, then filled with DMEM/10% FBS. Cells were kept at 37°C and 5% CO_2_ with a LiveCell system (Neue Biosciences, Camp Hill, PA). Fibroblast mobility was observed using a Nikon Eclipse TE2000U microscope (Nikon Instruments, Melville, NY) and MetaMorph software (Molecular Devices, Downington, PA) hourly over a course of 48 hours. Scratch closure was measured by measuring pixels of unfilled area and fibroblast area from images using Photoshop software (Adobe, San Jose, CA).

### Phagocytosis Assays

The derivation of bone marrow-derived macrophages (BMMs) was performed as previously described.[42] Briefly, macrophages were derived from the bone marrow of WT and TSP-1−/− mice using a 7-day course of MCSF in RPMI/5% FBS (Mediatech, Manassas, VA). To measure phagocytosis, we used a previously described protocol [30]. Briefly, cells were dissociated using sterile swabs and resuspended at a density of 1×10^6^ cells/mL in sterile PBS/0.5% FBS (Mediatech) and co-incubated with IgG-opsonized and PKH26 Red Fluorescent-labeled sheep red blood cells (SRBCs) for 1 hour at 37°C (Sigma-Aldrich, St. Louis, MO; Colorado Serum Company, Denver, CO). Cells were then washed and non-ingested SRBCs were lysed with hypotonic water. Samples were fixed in 1% formaldehyde and placed on microscope slides for blinded counting using an Olympus BX41 microscope and MicroSuite Special Edition software (Olympus America Inc, Center Valley, PA). Phase contrast pictures of BMMs were overlayed with fluorescent pictures showing ingested SRBCs using Adobe Photoshop software.

### Flow cytometry

Alveolar macrophages were collected from mouse lung BAL (n≥5 animals per group) and washed once in PBS. Cells were counted and equal numbers of cells were resuspended in flow cytometry buffer (1% BSA, 0.1% Sodium Azide in PBS). To block Fc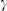 receptors I/II, cells were incubated with mouse CD16/32 IgG (BD Biosciences, San Diego, CA) on ice for 15 minutes then incubated with anti-CD64 conjugated to PE (BD Biosciences) on ice for 30 minutes. Cells that were immunostained with F4/80-APC conjugated antibody (AbD Serotec, Raleigh, NC), were subjected to a prior incubation with mouse IgG (Jackson ImmnuoResearch, West Grove, PA) to block Fc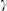 receptors. After immunostaining, cells were washed in flow cytometry buffer and fixed in 1% paraformaldehyde solution prior to flow cytometry analysis (LSRII; BD Biosciences). Data was analyzed using FCS3 Express software from De Novo Software (Los Angeles, CA). The proportion of CD64 and F4/80 positive cells are presented as the percent of all gated cells and are expressed as the mean ± SEM.

### Statistical Analysis

Survival studies were carried out to seven days post intervention and characterized by the methods of Kaplan-Meier. Differences in survival across genotypes (WT vs. TSP-1−/−) was compared using the log-rank test. Differences in colony forming units (CFU) across genotypes and time were tested using linear regression. Specifically we stabilized the variance of CFU across genotypes and normalized the distribution by using a cubed-root transformation and then regressed the dependent variable on time and genotype including their interaction. If this interaction was not significant, then it was dropped from the regression model. A two-sample *t*-test was used to compare the cubed-root of CFU between genotypes at 3 hours post CLP and to compare the phagocyctic index between genotypes. Delta cycle time from RT-PCR was used to compare expression levels CTGF mRNA, VEGF mRNA, and collagen 1 mRNA over genotype and time. Specifically ANOVA was used to determine if there was an overall difference in the delta cycle times. If this difference was significant, then a post hoc test comparing delta cycle times across genotypes for each time point after CLP was run. The *p*-values from the post hoc test were adjusted using the Holm's procedure to conserve the overall type I error at 0.05. This same procedure was used to compare active TGFβ1 and compare total collagen 1 across genotype and time. Random-effects linear regression was used to test if wound closure was different over genotype and time. Specifically we log transformed the wound size percentage and tested if the slopes (halving times) of each genotype was significantly different from zero (not decreasing with time) and test if the slopes were significantly different across genotypes. We also included an indicator variable that took into account early wound healing and late wound healing and tested these slope for each period. The cut point between early and late wound healing was determined post hoc after observing the graphed wound size percentage for each genotype. Finally, cytokine data in Table 2 were found to be non-normally distributed by the skewness and kurtosis test. As a result the Kruskal-Wallis test was used for analysis and data are now all represented as medians and IQR. All analyses were run on Stata 11.1, Stata Corporation, College Station, TX.
